# Comparison of outcomes for veterans receiving dialysis care from VA and non-VA providers

**DOI:** 10.1186/1472-6963-13-26

**Published:** 2013-01-18

**Authors:** Virginia Wang, Matthew L Maciejewski, Uptal D Patel, Karen M Stechuchak, Denise M Hynes, Morris Weinberger

**Affiliations:** 1 Health Services Research and Development, Durham VA Medical Center, Durham, NC, 27705, USA; 2 Division of General Internal Medicine, Department of Medicine, Duke University, Durham, NC, 27705, USA; 3 Division of Nephrology, Department of Medicine, Duke University, Durham, NC, 27705, USA; 4 VA Information Resource Center, Hines VA Hospital, Riverside, IL, 60546, USA; 5 Center for Management of Complex Chronic Care, Hines VA Hospital, Riverside, IL, 60546, USA; 6 Department of Medicine, University of Illinois-Chicago College of Medicine, Chicago, IL, 60612, USA; 7 Department of Health Policy and Management, School of Public Health, University of North Carolina at Chapel Hill, Chapel Hill, NC, 27599, USA

**Keywords:** Veterans, Dialysis, Hospitalization, Mortality

## Abstract

**Background:**

Demand for dialysis treatment exceeds its supply within the Veterans Health Administration (VA), requiring VA to outsource dialysis care by purchasing private sector dialysis for veterans on a fee-for-service basis. It is unclear whether outcomes are similar for veterans receiving dialysis from VA versus non-VA providers. We assessed the extent of chronic dialysis treatment utilization and differences in all-cause hospitalizations and mortality between veterans receiving dialysis from VA versus VA-outsourced providers.

**Methods:**

We constructed a retrospective cohort of veterans in 2 VA regions who received chronic dialysis treatment financed by VA between January 2007 and December 2008. From VA administrative data, we identified veterans who received outpatient dialysis in (1) VA, (2) VA-outsourced settings, or (3) both (“dual”) settings. In adjusted analyses, we used two-part and logistic regression to examine associations between dialysis setting and all-cause hospitalization and mortality one-year from veterans’ baseline dialysis date.

**Results:**

Of 1,388 veterans, 27% received dialysis exclusively in VA, 47% in VA-outsourced settings, and 25% in dual settings. Overall, half (48%) were hospitalized and 12% died. In adjusted analysis, veterans in VA-outsourced settings incurred fewer hospitalizations and shorter hospital stays than users of VA due to favorable selection. Dual-system dialysis patients had lower one-year mortality than veterans receiving VA dialysis.

**Conclusions:**

VA expenditures for “buying” outsourced dialysis are high and increasing relative to “making” dialysis treatment within its own system. Outcomes comparisons inform future make-or-buy decisions and suggest the need for VA to consider veterans’ access to care, long-term VA savings, and optimal patient outcomes in its placement decisions for dialysis services.

## Background

Approximately 35,000 veterans enrolled in the Veterans Health Administration (VA) have end-stage renal disease (ESRD), reflecting a higher prevalence in the VA population than in the general US population (604 vs. 187 per 100,000) [1,2] that is likely due to veterans’ older age and greater disease prevalence of diabetes and hypertension. The organization of chronic dialysis services for veterans with ESRD is complex because many are eligible for dialysis care through either the VA, the largest integrated healthcare system in the US, or the Medicare program.^a^ VA finances roughly 60% of ESRD dialysis services for these veterans and Medicare finances approximately 30% of dialysis services [3-5]. Veterans are likely to favor dialysis care through VA because the VA’s integrated care system provides a continuum of care and patients’ out-of-pocket cost-sharing is lower in VA than Medicare.^b^ To serve its ESRD veterans seeking dialysis care, the VA operates 74 hospital-based VA dialysis units that provide acute (inpatient) and chronic (outpatient) treatments. When the VA is not capable of meeting all veterans’ demand for chronic outpatient dialysis or providing timely geographic access to thrice weekly dialysis care at VA medical centers (VAMCs), the VA outsources dialysis care by purchasing services from non-VA providers in the private sector on a fee-for-service basis.

Prior work has examined utilization and outcomes of renal care in VA and Medicare systems [4,6], but less is known about the VA’s own organization of in- and out-of-network chronic dialysis treatment services. VA-outsourced dialysis is a major contributor to increasing ESRD costs for the VA. While overall outpatient dialysis expenditures for VA dialysis increased 10% between 1993 and 2003, payments for VA-outsourced dialysis grew 348% (from $13.5 M to $60.7 M) during the same period [7]. More recently, national expenditures for VA-outsourced care further increased to $432 M in 2011 [8]. Growth in VA dialysis spending is likely due to a combination of growth in the number of veterans with ESRD seeking VA coverage and increasing costs of dialysis treatment by outsourced providers. Of particular concern is the VA’s reliance on outsourcing dialysis services because VA exerts little clinical oversight or accountability for quality or outcomes of outsourced care furnished by non-VA providers.

Research has generally found outcomes of VA care to be as good or better than non-VA care [9]. The few studies comparing VA and non-VA (e.g., Medicare, Medicaid) systems for ESRD patient care are more mixed: renal patients obtaining care in VA receive earlier pre-dialysis nephrology care [10] and have better vascular access [11,12], but may have less access to kidney transplantation [13] than care received in non-VA settings.

In this study, we compare one-year hospitalizations and mortality of veterans with ESRD who receive dialysis through VA in-house and outsourced settings because no prior study has examined outcomes between these two settings. We also examine patient differences by dialysis setting to understand whether healthier or sicker veterans are more likely to be seen in outsourced settings. Compared to VA-outsourced care in the private sector, the VA’s closed system of care allows veterans to have multiple VA clinic visits that coincide with dialysis treatment sessions (3 times per week), which provides timely access to address critical health needs. Thus, we hypothesized that veterans receiving dialysis exclusively in VA settings had lower hospitalization and mortality rates than patients receiving dialysis in VA-outsourced providers in the private sector. Since dual use of VA and non-VA care may disrupt continuity of care and worsen health outcomes [14,15], we expected outcomes among veterans obtaining dialysis in both VA and VA-outsourced settings to be different than patients exclusively receiving either VA or VA-outsourced dialysis.

It is important to understand if outcomes differ between VA, VA-outsourced and dual system dialysis use, because VA expenditures for “buying” outsourced dialysis are high and increasing relative to “making” dialysis treatment within its own system and outcomes comparisons may inform future make or buy decisions. Such understanding is also important in other non-VA healthcare settings because make-buy planning decisions are a challenge currently faced by other large health systems and will grow as new care delivery models (e.g., Accountable Care Organizations) develop.

## Methods

### Study design and population

**Figure 1:**
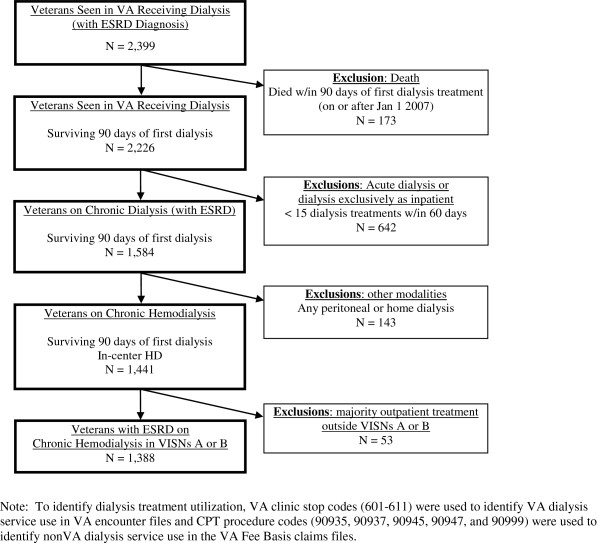
**Cohort of chronic dialysis veterans.** Note: To identify dialysis treatment utilization, VA clinic stop codes (601–611) were used to identify VA dialysis service use in VA encounter files and CPT procedure codes (90935, 90937, 90945, 90947, and 90999) were used to identify nonVA dialysis service use in the VA Fee Basis claims files.

We conducted a retrospective cohort study of veterans with ESRD in two regional, Veterans Integrated Service Networks (VISN) who received chronic outpatient dialysis treatment financed by VA between January 1, 2007 and December 31, 2008. To identify veterans with ESRD receiving chronic outpatient dialysis, our sampling strategy was adapted from the US Renal Data System (national repository of ESRD information) [16] for analysis using VA administrative data. We identified 2,540 veterans who received any dialysis services financed by VA either in VA or outsourced through the VA Fee Basis Program between January 2007 and December 2008 (Figure 1). We excluded patients who died within the first 90 days of their first treatment during the observation period (n=314) because dialysis treatment and exposure to treatment setting would be too limited to derive meaningful effects [1]. To further limit our examination of chronic dialysis patients receiving care in outpatient settings, we also excluded veterans who received: 1) only acute dialysis (defined as fewer than 15 dialysis treatments in a 60-day window) [16] or chronic dialysis exclusively as inpatients (n=642); 2) any home-based dialysis treatment (e.g., peritoneal dialysis, which is rarely used in the general US population and less so among the veteran population) (n=143); or 3) received the majority of their outpatient dialysis treatments outside the two study regions (n=53).

### Data and measurement

Our outcomes of interest were all-cause hospitalizations and mortality. We used the VA Patient Treatment (PTF) and Fee Basis (FB) files to identify hospitalizations and lengths-of-stay. All-cause mortality was identified from the VA Vital Status file. For all outcomes, we followed each subject one year from their start date which is defined as the first date of dialysis during the study period (i.e., on or after January 1, 2007).

VA outpatient encounter and FB claims files were used to determine each veteran’s start date and to identify veterans’ source of outpatient dialysis treatment thereafter. Outpatient dialysis treatment setting was the explanatory variable of interest, defined as either exclusively VA; exclusively VA-outsourced dialysis, or dual settings (defined as receiving at least one outpatient-based dialysis treatment in VA and VA-outsourced dialysis).

We generated several baseline patient covariates. Following VA convention [17], we assessed the most recently reported race (e.g., White, non-White, or unknown) from VA inpatient and outpatient encounter data. We obtained date of birth and gender from the VA Vital Status File, and we determined veteran eligibility and copayment status from the VA Enrollment file. Comorbidity burden for each veteran in the year prior to baseline was assessed using the Diagnostic Cost Group (DCG) risk score from the annual VA DCG file, which is as predictive of veterans’ one-year mortality and expenditures as other comorbidity scores [18-20]. We used PTF and FB files to determine whether veterans were hospitalized in the year prior to baseline. We also determined each patient’s history of dialysis use prior to each subject’s start date in four mutually exclusive time periods: none (i.e., incident users), 6 months of prior use, 1 year of prior use, and 2 years of prior use. Distance to VA dialysis was defined as straight-line distance (in miles) between veterans’ residence zip code and the nearest VA dialysis unit zip code centroid. Last, we included a dichotomous indicator of VISN to control for patients’ service region effects.

### Analysis

To examine whether healthier or sicker veterans are more likely to be seen in outsourced settings, we compared patient characteristics and unadjusted outcomes across veterans in the three dialysis settings using Chi-square tests for categorical variables and ANOVA tests for continuous variables. We then compared adjusted one-year outcomes using multivariable regression models. For our hospitalization outcome, we conducted logistic regression to estimate the probability of hospitalization at one year, and then conducted negative binomial regression to examine total hospitalizations and cumulative inpatient length-of-stay among the subset of veterans who were hospitalized. One-year mortality was analyzed via logistic regression because we were not able to observe patients’ disease start or first ever dialysis treatment for assessing time-to-death. All models controlled for veterans’ age, sex, race, marital status, copayment status, distance to nearest VA dialysis unit, history of dialysis utilization, a dichotomous indicator of prior hospitalization, comorbidity burden measured by the DCG score, and veterans’ Integrated Service Network of residence. To determine whether mortality differences across dialysis settings varied on the basis of hospitalization during the follow-up period, we also conducted stratified analyses of mortality among veterans who were hospitalized at least once in the year after baseline and veterans who had no hospitalizations. This study and requisite waivers of patient informed consent for retrospective analysis of administrative data were approved by the Institutional Review Board of the Durham VA Medical Center.

## Results

### Descriptive statistics

The final analytic cohort included 1,388 veterans who received chronic outpatient hemodialysis treatment financed by VA (Figure 1): 381 (27%) received dialysis exclusively in the VA, 659 (47%) exclusively received VA-outsourced dialysis, and 348 (25%) were dual users (Table 1). VA users were more likely to be non-White, unmarried, younger, had higher comorbidity burden, and lived closer to the nearest VA dialysis unit than veterans exclusively receiving outpatient dialysis in VA-outsourced or in dual dialysis settings (all, *p*<0.01).

**Table 1:** VA chronic dialysis patient baseline characteristics, by outpatient dialysis setting

	**Overall (n=1,388)**	**VA (n=381)**	**Non-VA (n=659)**	**Dual (n=348)**	***p***
Age	62.2 (11.3)	61.7 (11.5)	63.2 (11.4)	61.0 (11.0)	.005
Male, %	98.6	99.0	98.6	98.3	.736
Race, %					<.001
White	38.3	24.7	47.6	35.3	--
Non-White	59.0	74.0	47.5	64.4	--
Unknown	2.7	1.3	4.9	0.3	--
Marital status, %					<.001
Married	47.0	38.1	53.1	45.4	--
Unmarried	50.9	61.2	43.7	53.4	--
Unknown	2.0	0.8	3.2	1.1	--
Copayment required, %	6.0	8.1	5.3	4.9	.110
Diagnostic Cost Group (DCG) score	5.1 (3.1)	5.7 (3.1)	4.5 (3.0)	5.7 (3.0)	<.001
Arrhythmia, %	15.9	19.4	14.7	14.1	.078
Cancer, %	12.5	13.1	10.2	16.1	.023
Cerebrovascular disease, %	13.4	13.4	12.4	15.2	.467
Congestive heart failure, %	28.2	28.3	28.2	28.2	.998
Chronic pulmonary disease, %	16.9	15.2	17.8	17.2	.568
Diabetes, %	63.8	60.9	62.5	69.5	.033
Hypertension, %	87.2	85.3	85.0	93.4	<.001
Myocardial infarction, %	6.3	5.2	7.0	6.3	.544
Liver disease, %	6.0	6.0	5.0	7.8	.216
Peripheral vascular disease, %	20.7	27.3	17.0	20.7	<.001
History of dialysis prior to baseline, %					.001
None	48.6	51.4	43.1	56.0	--
6 months	8.2	5.5	9.7	8.3	--
1 year	5.8	4.7	6.4	5.8	--
2 years	37.4	38.3	40.8	29.9	--
History of hospitalization 1-year prior to baseline, %	52.2	63.5	41.6	59.8	<.001
Home VA station has dialysis, %	66.2	100	35.7	87.1	<.001
Distance to home VA station, miles	35.5 (45.4)	12.4 (39.7)	46.2 (38.6)	40.5 (53.5)	<.001
Distance to nearest VA dialysis, miles	58.6 (59.6)	12.1 (36.4)	91.2 (52.0)	48.0 (55.6)	<.001
Region, %					<.001
VISN A	73.7	51.2	83.5	79.9	--
VISN B	26.3	48.8	16.5	20.1	--
All-cause Hospitalization, %	47.9	65.1	30.2	62.6	<.001
Hospitalizations for all subjects, mean	1.2 (1.7)	1.7 (2.0)	0.6 (1.3)	1.6 (2.0)	<.001
Hospitalizations for patients with any hospitalization^3^, mean	2.4 (1.8)	2.6 (1.9)	2.1 (1.5)	2.6 (1.9)	.002
Total length of stay for all subjects, mean	10.0 (27.1)	14.9 (32.9)	5.0 (15.0)	14.0 (35.3)	<.001
Total length of stay for patients with any hospitalization^3^, mean	20.9 (36.2)	22.9 (38.5)	16.6 (23.6)	22.4 (42.4)	.146
All-cause Mortality, %	12.5	14.7	13.4	8.3	.022

1. Notes: 1. Mean (SD) reported, unless noted otherwise.

2. ANOVA and Chi-square tests for differences by user type.

3. Reported means and standard deviations among patients with any hospitalization (n= 655, 47.9%).

### Hospitalization differences between VA, VA Fee dialysis and dual users

Approximately half (48%) of the cohort was hospitalized one year after veterans’ baseline dialysis visit (Table 1). Veterans receiving dialysis exclusively in VAMCs were more likely to be hospitalized, compared to dual and VA-outsourced dialysis users (65%, 30%, and 63% respectively, *p*<0.001). Among those with any hospitalization (n=665), veterans receiving dialysis in VA settings incurred more all-cause hospitalizations (2.6 vs. 2.1, p<0.01) than VA-outsourced dialysis users. Veterans receiving dialysis in VA had greater cumulative lengths-of-stay on average (22.9) than those in VA-outsourced or dual settings (mean= 22.4 vs. 16.6 days, respectively), though these differences were not statistically significant (*p*=0.146).

Consistent with the unadjusted results, veterans who received outpatient dialysis exclusively in VA-outsourced settings were less likely to be hospitalized than VA dialysis users (OR= 0.35, *p*<0.001, Table 2). Among the hospitalized veterans in our cohort, patients receiving VA-outsourced dialysis incurred fewer inpatient admissions (β= −0.16, *p*<0.05) and had shorter total length-of-stay than patients receiving VA dialysis (β= −0.37, *p*<0.05). Higher comorbidity burden and hospitalization in the year prior to baseline was positively associated with any hospitalization and the number of hospitalizations (*p*<0.01).

**Table 2:** Adjusted all-cause hospitalization 1-year after baseline

	**Any Hospitalization (n=1,388)**	**Total Number of Hospitalizations (n=665)**^**3**^	**Total Hospital Length of Stay (n=665)**^**3**^
	**OR**	**β**	**β**
Dialysis setting: Non-VA (ref: VA)	.35*** (.24, .51)	–.16* (.08)	–.37* (.15)
Dual	.99 (.70, 1.40)	.01 (.07)	–.05 (.15)
Age	1.00 (.99, 1.01)	–.01* (.003)	–.01 (.004)
Male	.96 (.36, 2.57)	–.08 (.25)	.01 (.42)
Race: Non-White (ref: White)	.90 (.68, 1.18)	–.07 (.06)	–.17 (.13)
Unknown	.20** (.07, .57)	–.06 (.27)	–.86* (.39)
Marital status: Unmarried (ref: married)	1.15 (.89, 1.49)	.05 (.05)	–.17 (.13)
Unknown	.64 (.26, 1.56)	.15 (.20)	–.15 (.32)
Copayment required	.90 (.55, 1.48)	–.11 (.11)	.33 (.37)
Diagnostic Cost Group^4^	1.22*** (1.17, 1.27)	.03** (.01)	–.01 (.02)
History of dialysis prior to baseline: 6 months (ref: none)	.62* (.39, .99)	–.15 (.10)	–.18 (.14)
1 year	.27*** (.16, .47)	–.13 (.12)	–.15 (.26)
2 years	.43*** (.33, .57)	.01 (.06)	.08 (.11)
History of hospitalization 1-year prior to baseline	2.13*** (1.65, 2.74)	.28*** (.06)	.28* (.13)
Distance to nearest VA dialysis, miles	.999 (.997, 1.002)	–.0005 (.0004)	–.0005 (.001)
Region: VISN B (ref: VISN A)	1.59** (1.18, 2.13)	.08 (.06)	–.14 (.13)
C-statistic	0.79	–	–

Notes:

1. * p< .05 **p< .01 ***p<.001.

2. Statistics reported in parens reflect robust 95% Confidence intervals for odds ratio and standard errors for beta coefficients.

3. Total hospitalizations and hospital lengths of stay were modeled with the subset of patients who experienced any hospitalization (n= 655).

4. Results from a restricted model using DCG comorbidity adjustment are shown here. Models that included individual comorbidity indicators yielded similar results.

### Mortality differences between VA, VA Fee dialysis and dual user

Unadjusted one-year mortality was greater among VA users (15%) compared to VA-outsourced dialysis patients (13%) and dual system dialysis users (8%, *p*<0.05). Compared with VA dialysis (Table 3), use of both systems for outpatient dialysis was associated with lower mortality one-year after Veterans’ baseline (OR=0.52, *p*<0.05).

**Table 3:** Adjusted all-cause mortality 1-year after baseline

	**Overall (n=1,388)**	**Stratified Sample**^**3**^	
		**No Hospitalization (n=723)**	**≥ 1 Hospitalization (n=665)**
Dialysis setting: Non-VA (ref: VA)	.80 (.48, 1.33)	2.93 (.99, 8.64)	.79 (.45, 1.39)
Dual	.52* (.31, .86)	1.40 (.40, 4.90)	.42** (.24, .74)
Age	1.05*** (1.03, 1.06)	1.05*** (1.03, 1.08)	1.05*** (1.03, 1.07)
Male	2.30 (.29, 18.54)		
Race: Non-White (ref: White)	.66 (.46, .96)		
Unknown	.96 (.40, 2.32)		
Marital status: Unmarried (ref: married)	1.34 (.95, 1.89)		
Unknown	1.22 (.43, 3.43)		
Copayment required	1.21 (.65, 2.27)		
Diagnostic Cost Group	1.04 (.98, 1.10)	1.03 (.93, 1.15)	.99 (.92, 1.06)
History of dialysis prior to baseline:			
6 months (ref: none)	.84 (.46, 1.55)	1.35 (.53, 3.45)	.65 (.28, 1.51)
1 year	.61 (.27, 1.39)	.69 (.20, 2.38)	.76 (.26, 2.23)
2 years	.92 (.64, 1.33)	1.03 (.55, 1.90)	1.07 (.65, 1.74)
History of hospitalization 1-year prior to baseline	1.30 (.90, 1.8)	1.01 (.56, 1.84)	1.30 (.82, 2.05)
Distance to nearest VA dialysis, miles	1.001 (.997, 1.004)	1.00 (.99, 1.01)	1.00 (1.00, 1.01)
Region: VISN B (ref: VISN A)	.94 (.63, 1.40)		
C-statistic	0.70	0.71	0.70

1. Notes: 1. * p< .05 **p< .01 ***p<.001.

2. Adjusted odds ratios reported with robust 95% Confidence intervals noted in parens.

3. Due to the limited distribution of deaths in these stratified samples, explanatory variables in the stratified mortality analyses were restricted to several key covariates, shown above. Estimates from this restricted model did not differ from estimates from the unrestricted model.

In mortality analysis stratified by hospitalization to examine whether the association between dialysis setting and mortality was moderated by hospitalization, we restricted the explanatory variables to dialysis setting, age, comorbidity burden, dialysis utilization and hospitalization prior to baseline, and distance to nearest VA dialysis unit. We found no statistically significant differences in mortality by dialysis setting among veterans who were not hospitalized (*p*=0.05-0.60, Table 3 columns 2–3). For those who were hospitalized, dual users had lower one-year mortality than VA-only dialysis patients (OR=0.42, *p*<0.01).

## Discussion

In this study, veterans receiving outpatient chronic dialysis services from VA-outsourced dialysis providers were less likely to be hospitalized and had shorter lengths-of-stay when hospitalized, compared to veterans receiving dialysis exclusively in VA. These outcome differences are likely due to favorable selection into VA-outsourced dialysis because VA-outsourced dialysis veterans were generally healthier than those who either used VA services exclusively or used both systems for dialysis care. Since our adjusted results accounted for comorbidity burden, there may be other explanations for our findings. For example, it is likely that VA retains the sickest patients and triages lower risk patients outsourced dialysis care. It is also possible that higher hospitalization rates of VA dialysis users may be attributed to unmeasured factors or factors unrelated to ESRD or dialysis treatment. Alternatively, VA-outsourced dialysis facilities in the private sector may provide better care than VA, despite inducing greater care fragmentation. This latter explanation contradicts findings from Trivedi and colleagues’ [9] review which found outcomes in most primary care contexts to be generally better in VA than non-VA settings. Considering that chronic dialysis patients require frequent, costly, and life-sustaining treatment that places them at high risk of adverse events (e.g., cardiac events, infection), further research on the quality of VA and VA-outsourced dialysis-specific care is needed.

There were no mortality differences between exclu-sive use of dialysis services through the VA or VA-outsourced dialysis settings. Dual dialysis users had lower one-year mortality than VA dialysis users, which is largely driven by the 48% of veterans who were hospitalized within one-year after baseline (OR=0.42, *p*<0.01). Even though VA-only and dual-system dialysis patients had similar baseline comorbidity burden, dual-system dialysis users may have lower one-year mortality because they were healthy enough to have their care outsourced to a non-VA provider in the community during the year of follow-up.

Interestingly, one-year mortality in this veteran cohort was lower than that of the general ESRD population that received dialysis care from Medicare [16]. These findings were consistent with recently reported [4] one-year mortality of veterans receiving chronic dialysis treatment in VA and Medicare programs. The relatively low mortality among veterans receiving VA-financed dialysis may reflect favorable pre-dialysis care among VA and dual use patients observed in several other studies [10-12], or veterans’ access to comprehensive, non-dialysis medical care concurrent with thrice weekly hemodialysis treatment.

The demographic characteristics, mortality rates, and hospitalization rates in our two region, 2007–2008 cohort were similar to characteristics of dialysis patients from 2001–2003 examined by Hynes and colleagues (2012). However, the dialysis usage patterns are quite different across the two studies: Hynes et al. found that 50% of dialysis patients dialyzed in VA (versus 27% in this study) and 11% received VA-outsourced dialysis (versus 47% here). These usage differences may be due to the different sampling approaches, because the Hynes study prospectively recruited and followed a smaller sample of patients (n=334) for primary data collection, whereas here we retrospectively sampled a larger sample through administrative data. These differences in the two studies’ geographic sampling and the regional differences we found in our own sample suggest variations that may be due to disease prevalence, local in-house treatment capacity, or local interpretation and practices of system-wide level policies. It is imperative to explore this variation at a larger scale to assist VA ensure dialysis supply and quality of care for its patients, system-wide.

It behooves VA policymakers in the health system to carefully consider options to address its system of care for a growing VA patient population with ESRD. Based on our results, VA’s use of outsourced dialysis in communities may be an appropriate solution that allows VA to free up limited capacity in existing VA units to treat acute and sicker chronic patients and minimize patients’ risk for hospitalization. Since VA patients had a nearly significant, three-fold adjusted risk of death, VA will need to pay close attention to care coordination and oversight of veterans receiving outsourced dialysis. Alternatively, if VA-outsourced dialysis veterans are systematically less complex, then building additional VA capacity and patient access to more VA-based dialysis may improve continuity of VA care and reduce VA expenditures on outsourced dialysis care without compromising patient survival outcomes. Another approach is to formally establish a system of dual-dialysis, where veterans start dialysis in VA and later transition to VA-outsourced settings once they are healthy enough for care in the community. Further research on the impact of dual use (e.g., sequencing of service settings and care transitions) on outcomes are needed to inform the merits of this dual-system approach. Ultimately, the tradeoffs associated with “making” more VA dialysis must be balanced against the tradeoffs of “buying” outpatient dialysis services from VA-outsourced providers and potential savings in VA dollars related to other medical care.

The challenges of striking the optimal balance of supply, access, quality, and costs for dialysis are not unique to the VA: the development of large integrated health systems and advent of accountable care organizations (ACOs) [21,22] emphasize the need to reorganize models of care delivery to address the same challenges. As the largest integrated healthcare system in the United States, the VA is an ACO that is directly responsible for providing comprehensive health services, ensuring coordinated and high quality care among providers that are accountable to each other, while keeping down costs. In this way, lessons learned from the VA’s efforts to reorganize life-sustaining dialysis care, as a component of comprehensive patient care for those with ESRD, may inform strategies to improve chronic disease care delivery in ACOs and other health systems.

### Limitations

There are a number of study limitations that should be noted. First, the scope of our analysis was limited to outpatient dialysis utilization and hospitalizations provided under financial auspices of the VA because we did not have access to administrative data on Medicare-funded services or patients’ other forms of health insurance coverage at the time of this study. While the VA is the likely principal source of healthcare for veterans in our study due to the high frequency of VA copayment exemption (94%) that favors VA- over Medicare-financed dialysis care (no copayment under VA coverage versus 20% copayment under Medicare), most ESRD veterans are eligible for Medicare as an alternative to VA coverage [3,4,16]. We therefore cannot discount patients’ use of other medical care in non-VA settings: to the extent that veterans were hospitalized under Medicare coverage, the lower hospitalization rate among VA-outsourced patients may be due to Medicare-covered admissions within the one-year follow-up period that were not observable in the VA administrative files.

Second, we were not able to examine intermediate outcomes of dialysis treatment (e.g., urea reduction ratio, anemia treatment.), that reflect the quality of dialysis care across settings and explain differences in outcomes. Clinical measures like VA laboratory results are available in veterans’ VA electronic health record, but outsourced dialysis providers do not report comparable information on its veteran patients to VA. Third, we were not able to identify or characterize VA-outsourced dialysis providers from the VA fee basis claims data. This is important because the majority of private-sector dialysis facilities are freestanding units and studies that found relationships between ESRD outcomes and dialysis facility characteristics have excluded VA’s hospital-based facilities [23,24]. Further research is needed to better understand whether observed differences in veterans’ dialysis outcomes are attributable to the types of providers VA is outsourcing its services.

Fourth, our sampling frame included prevalent and incident ESRD patients for whom we were not able to observe ESRD vintage (e.g., time since ESRD diagnosis or first dialysis treatment), which may be positively associated with assigned treatment setting, hospitalization, and mortality. To minimize the impact of this potential confounder, we developed a proxy of duration on dialysis (dialysis 0 – 2 years before each subject’s study start date) for adjusted analyses. Last, we sampled patients from two VA service regions which illustrated variation in the use of treatment settings, but constituted a small sample of subjects and occurrences of outcomes to analyze via unrestricted, adjusted cause-specific hospitalization and mortality models. Despite our efforts to address confounding and selection biases, these elements of our study design complicate translation of our results. Thus, our findings should be interpreted with caution. Future research with a nationally representative sample of patients’ and their disease and treatment history will enable survival analysis to assess outcomes with better precision.

## Conclusions

This is the first study to compare hospitalizations and mortality associated with veterans receiving VA-financed and coordinated dialysis treatment services in VA, VA-outsourced, and dual settings. Veterans receiving non-VA dialysis are systematically less complex, less likely to be hospitalized, and had shorter lengths-of-stay than Veterans receiving VA dialysis. For dual dialysis patients, the likelihood of hospitalization is similar but mortality is reduced. These findings suggest the need for VA to consider Veterans’ access to care, long-term savings in VA expenditures, and optimal patient outcomes in its strategy for delivering dialysis services. As VA considers new ways to efficiently and effectively reorganize dialysis care to address critical shortages in service capacity for a growing veteran population with ESRD, this study provides an important step in continued assessment of dialysis outcomes to inform the impact of its make-buy and care management decisions for dialysis services.

## Endnotes

^a^Veterans may not be able to obtain financial coverage through both the federal VA healthcare system and Medicare programs, simultaneously. VA coverage is variable because receive a graded amount of benefits based on armed service-connection status and income. Veterans who have not previously qualified for Medicare by age or disability status are eligible for Medicare if they develop renal failure and have worked the required amount of time to be eligible for Social Security benefits, but must wait 90-days to enroll in the Medicare ESRD program and another 33-months for Medicare to serve as the primary payor for healthcare services.

^b^Patient cost-sharing is $0 or $15 per treatment session for veterans financing care through VA versus Part B premiums and 20% copayment of charges (approximately $30 per treatment) under Medicare.

## Abbreviations

ACO: Accountable Care Organization; DCG: Diagnostic Cost Group; ESRD: End Stage Renal Disease; FB: Fee Basis; PTF: Patient Treatment File; VA: Veterans Health Administration; VAMC: Veterans Affairs Medical Center; VISN: Veterans Integrated Service Network.

## Competing interests

Dr. Maciejewski reports serving as consultant for Takeda Pharmaceuticals, Novartis, and the Surgical Review Corporation and owning stock in Amgen; his spouse is employed by Amgen. Dr. Patel has received consultation funds from Kai Pharmaceuticals, Abbott Laboratories, and received research funding from Amgen, Eli Lilly & Co., and Daiichi Sankyo for clinical trial event adjudication activities. The other authors (VW, DMH, KMS, MW) report no relationship or financial interest with any entity that would pose a conflict of interest to the subject matter of this article. The views expressed in this article are those of the authors and do not necessarily reflect the position or policy of the Department of Veterans Affairs, Duke University, University of Chicago, or the University of North Carolina at Chapel Hill. An earlier version of this manuscript was presented at the 2012 AcademyHealth and American Society of Nephrology Annual Research Meetings.

## Authors’ contributions

VW conceived and obtained funding of this study, led its design and statistical analysis, coordinated and drafted the manuscript. MLM, MW, and UDP participated in the study design, analysis and interpretation of data, and critical revision of the manuscript. DMH participated in the acquisition and analysis of data and provided critical revision of the manuscript. KMS performed data programming, conducted statistical analysis and drafted the manuscript. All authors read and approved the final manuscript.

## Pre-publication history

The pre-publication history for this paper can be accessed here:

http://www.biomedcentral.com/1472-6963/13/26/prepub
